# Indirect food web interactions mediated by predator–rodent dynamics: relative roles of lemmings and voles

**DOI:** 10.1098/rsbl.2013.0802

**Published:** 2013-12-23

**Authors:** Rolf A. Ims, John-André Henden, Anders V. Thingnes, Siw T. Killengreen

**Affiliations:** Department of Arctic and Marine Biology, University of Tromsø, Tromsø 9037, Norway

**Keywords:** predation, alternative prey mechanism, dummy nests, functional traits

## Abstract

Production cycles in birds are proposed as prime cases of indirect interactions in food webs. They are thought to be driven by predators switching from rodents to bird nests in the crash phase of rodent population cycles. Although rodent cycles are geographically widespread and found in different rodent taxa, bird production cycles appear to be most profound in the high Arctic where lemmings dominate. We hypothesized that this may be due to arctic lemmings inducing stronger predator responses than boreal voles. We tested this hypothesis by estimating predation rates in dummy bird nests during a rodent cycle in low-Arctic tundra. Here, the rodent community consists of a spatially variable mix of one lemming (*Lemmus lemmus*) and two vole species (*Myodes rufocanus* and *Microtus oeconomus*) with similar abundances. In consistence with our hypothesis, lemming peak abundances predicted well crash-phase nest predation rates, whereas the vole abundances had no predictive ability. Corvids were found to be the most important nest predators. Lemmings appear to be accessible to the whole predator community which makes them particularly powerful drivers of food web dynamics.

## 1. Introduction

Multiannual production cycles in birds are classic cases of indirect food web interactions driven by predator–prey dynamics [1,2]. The focal indirect interaction is mediated by an ‘alternative prey-mechanism’, whereby predators becoming abundant after abundance peaks in cyclic rodent populations (main prey) shift their predation to bird nests (alternative prey) during the subsequent crash phase [3,4]. Such rodent-driven interaction cycles have been demonstrated in different ecosystems [3–6]. However, the most profound bird production cycles have been reported from high-Arctic ecosystems, where the contrast in nest predation rate between cyclic phases may be extreme [7,8].

The strength of the alternative prey mechanism is likely to be determined by the species-specific traits of the prey and predator that are involved [2,3]. Here, we focus on the species within the rodent community. While many Arvicoline rodents (i.e. voles and lemming) exhibit multiannual cycles, different species reside in different ecosystems. Lemmings are the only species present in high-Arctic tundra, while voles dominate in the boreal forest [7,9]. As bird production cycles are most profound in high-Arctic tundra, we hypothesized that lemmings are particularly prone to drive strong indirect interactions with ground nesting birds.

The rodent community in low-Arctic tundra of northeast Norway consists of a mix of one lemming (Norwegian lemming *Lemmus lemmus*) and two vole species (grey-sided vole *Myodes rufocanus*, and tundra vole *Microtus oeconomous*) with similar abundances. Thus, this ecosystem provides an excellent case for testing the relative roles of the two groups of rodents in the indirect food web interactions involving ground nesting birds. Exploiting the occurrence of spatially variable cyclic peak abundances in the three rodent species, we show by means of dummy bird nests that the lemming was the key driver of the alternative prey mechanism.

## 2. Material and methods

**Figure 1.**
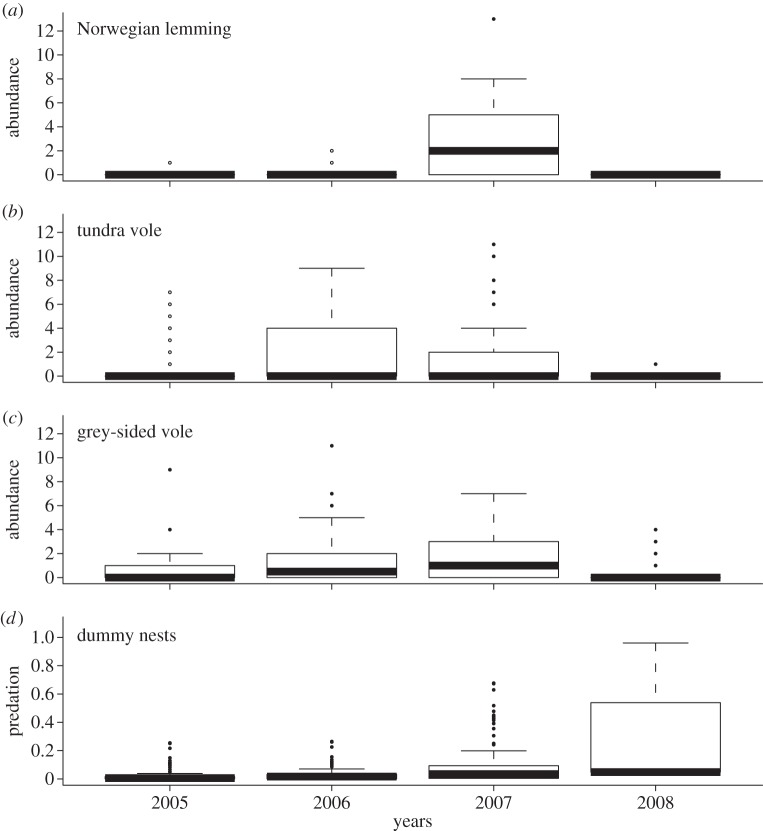
Box plots of (*a*–*c*) species-specific rodent abundance and (*d*) predation on dummy nest over the 4 year study period. The abundance of rodents is displayed as the number of individuals trapped per site (one individual per site corresponds to 4.17 individuals per 100 trap-nights).

The study was conducted over four summers (2005–2008) in eastern Finnmark (70–71° N). Rodent abundance was indexed at 74 widely spaced sites across a large region (i.e. spanning a distance of 100 km) and distributed equally among two common tundra habitats; dwarf-shrub heaths and riparian meadows interspersed with willow thickets [10]. At each site, a 15 m × 15 m rodent trapping unit was deployed [11] and operated for two trap-nights during each census. The trapping showed that the study period included the late increase (2005–2006), peak (2007) and crash phase (2008) of a 5 year cycle. As shown by previous analyses [11,12], the population crashes were spatially and interspecifically synchronous across the entire study region (figure 1), whereas the cycle amplitude (i.e. the peak abundances) exhibited substantial spatial variability within the species.

To assess predation risk, two dummy nests with one brown hen egg, similar in appearance to waterfowl eggs, were deployed per trapping unit and inspected at 5 day intervals during 15 days in early July. In the heath habitat, eggs were placed in a coup-sized pit in the short vegetation, whereas in the thicket habitat eggs were placed on a track-board [13] under the cover of tall willow shrubs. The track-board allowed for identification of predator species. Note that owing to the confounding between nest design and habitat, we do not intend to infer habitat-specific predation risk. Moreover, as dummy nests may not be representative of predation rates of natural nests, we focus on the variation in *relative predation risk* similar to previous studies of patterns of nest predation [14].

We use logistic mixed effects models (lmer function in package Lme4 in R) to analyse variation in nest predation rates among the 74 study sites. According to the alternative prey mechanism, we predicted predation to be highest in the crash phase of the rodent cycle; i.e. when predators are expected to switch prey from rodents to bird nests. Moreover, we predicted predation rates to increase with increasing preceding rodent abundances, because predators respond numerically to rodents with a 1 year time delay [15]. Consequently, we used the site-specific autumn rodent abundances in year *t* − 1 as predictors of nest predation rates in year *t*. As we target the relative impacts of voles and lemmings, we kept the three rodent predictors in all candidate models. As optional fixed effect covariates, we tested habitat (heath and thickets) and nest inspections (1–3) and their interactions with the rodent predictors. The best model structure was selected by log-likelihood ratio tests. To correct for the repeated measurements over sequential nest inspections and years, we included site as a random effect. Year was also added as a term to the best model to test whether the effect of rodent density had a significant spatial component. Finally, absence of positive spatial autocorrelation in the residuals from the best model was verified by computing Moran *I* statistics and plotting spline correlogramme (package spdep in R).

## 3. Results

**Figure 2.**
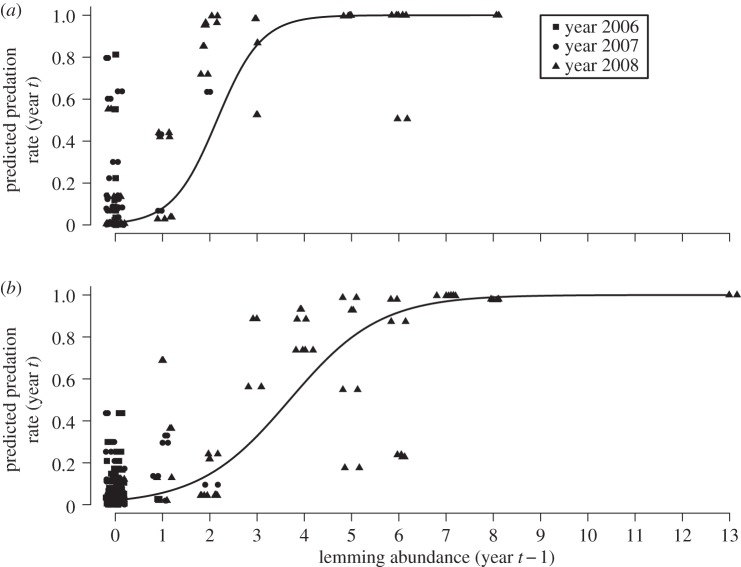
Predation rates in year *t* predicted by lemming abundances in year *t* − 1 in (*a*) thicket and (*b*) heath habitats (logit-slopes: *β*_thicket_ = 2.12, 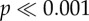, *β*_heath_ = 1.01, 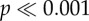). Predictions are shown for the first inspection of the nests in the season, but were qualitatively similar for all inspections. Year-specific predicted values are shown to highlight the spatial and temporal components of the estimated relation.

As expected, the nest predation rates peaked in the rodent crash year (2008; figure 1) although with substantial spatial variability among the sites (figures 1 and 2). None of the two vole predictors had any effect (*p* > 0.60), while the predation rates increased steeply with previous year lemming abundance (figure 2). The lemming effect interacted with habitat (logit-contrast of slope parameters: *β*_heath_ − *β*_thickets_ = −1.08, *p* = 0.003) with the highest predation rates on the track-boards in the thicket habitat (figure 2). There was also some evidence for an effect of inspection sequence (*β*_first_ − *β*_second_ = −0.601, *p* = 0.087; *β*_first_ − *β*_third_ = −1.18, *p* = 0.073). The negative effect of previous year lemming density on nest predation risk was still highly significant 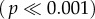 when year was added to the best model meaning that the lemming effect had a strong spatial component. The residuals from the best model exhibited a weak tendency for negative autocorrelation (Moran *I* = −0.0002, *p* = 0.031).

Corvids, and in particular ravens (*Corvus corax*), were responsible for most incidents with identifiable predator species on the track-boards (table 1).

**Table 1.** Identity of predators involved in predation events on track-boards.

	no. of events				
year	raven (*Corvus corax*)	hooded crow (*Corvus cornix*)	birds (undetermined)	red fox (*Vulpes vulpes*)	mustelids
2006	0	0	0	5	0
2007	10	0	5	0	0
2008	16	7	11	1	3

## 4. Discussion

The results were consistent with our hypothesis that the particularly strong and community-wide production cycles in high-arctic waders and waterfowl may be enforced by some characteristics of lemmings that make them stronger drivers of the alternative prey mechanism than voles. Lemmings have been proposed to possess several behavioural and demographic traits that make them more vulnerable to predators than voles [9]. For instance, lemmings are clumsier and appear to be more exposed during movements and foraging than voles. This proposal is consistent with a contemporary study of red fox diets in the same region that showed a strong selection for lemming [16]. The identity of the predators revealed by the track-boards in this study indicated that corvids were the most prevalent egg predators—a finding consistent with a quantitative survey of the predator community in the study region [17]. While corvids previously have been shown to be efficient predators of lemmings [18], it is a new finding that corvids respond locally to high lemming density in terms of a distinctly time-lagged switch to predation on bird eggs. This implies that omnivorous birds (e.g. corvids) may play a more important role in the dynamics of Arctic food webs than previously acknowledged. Future studies should elucidate to which extent behavioural (i.e. functional response and learning) and/or demographic responses drive corvid responses to lemming spatio-temporal dynamics in tundra ecosystems.

Recent studies have shown that lemmings appear to be functionally more important than voles in plant–herbivore interactions [19,20] with ramifications for indirect food web interactions mediated by plants [21]. Our study suggests that lemmings also are key drivers of indirect food web interactions mediated by predators. This may be so because lemmings are more accessible to the entire predator community than other prey species. Indeed, lemmings appear to possess a suite of functional traits that make them prime candidates of key-stone species for the ecological literature [22]. In light of their important function in the Arctic ecosystems, it is worrisome that lemmings appear to be particularly vulnerable to climate warming [12]. This vulnerability is now regionally expressed as severely dampened or entirely collapsed lemming cycles [23–25] with the expected knock-on on predators [26] and their alternative prey [27].

## Notes

The study was coordinated with the Directorate of Nature Management; which is the agency that authorises the specific methodologies applied in Norway.

## Data accessibility

Data available from the Dryad Digital Repository: http://dx.doi.org/10.5061/dryad.df119.
